# Process Evaluation of Digital Mental Health Interventions for Psychosis: Scoping Review With Framework Synthesis

**DOI:** 10.2196/88032

**Published:** 2026-07-23

**Authors:** Chloe Hampshire, Charlotte Dack, Shadi Daryan, Carolina Fialho, Rayan Taher, Ashley-Louise Teale, Jenny Yiend, Pamela Jacobsen

**Affiliations:** 1 Department of Psychology University of Bath Bath, England United Kingdom; 2 Institute of Psychiatry, Psychology & Neuroscience King's College London London, England United Kingdom

**Keywords:** digital mental health interventions, framework synthesis, process evaluation, psychosis, randomized controlled trial, scoping review

## Abstract

**Background:**

Implementing digital mental health interventions (DMHI) for those with psychosis is a persistent challenge. A process evaluation, or studies conducted alongside trials, is one research method that may address this issue. However, a synthesis of process evaluation data in this area is missing.

**Objective:**

This study aimed to understand what is known about context, implementation, and mechanisms of impact by synthesizing process evaluation data from trials evaluating DMHIs used by people with psychosis.

**Methods:**

A scoping review using a 2-phase search strategy underpinned by the Medical Research Council (MRC) process evaluation framework was conducted. Database searches of Cochrane Central Register of Controlled Trials and PsycInfo in 2024 and 2025 first identified an index sample of peer-reviewed trials predominantly conducted in the United Kingdom (≥50% of samples from the United Kingdom in multicountry studies). Next, papers linked to the index sample were retrieved and included if they reported process evaluation data as operationalized in the MRC framework. Two authors independently screened references, extracted summary data, and assessed the quality of index trials. One author qualitatively synthesized process evaluation data using a deductive framework synthesis approach using the MRC framework. Findings were triangulated with senior authors and presented as a narrative synthesis.

**Results:**

Searches identified 14 DMHIs and 45 papers reporting process evaluation data, though only 2 were labeled as such. Qualitative syntheses of process evaluation data generated five themes aligned with the MRC framework: (1) enhancing fit and supporting delivery (implementation strategies); (2) DMHI implementation varied across users, staff, and delivery settings (implementation outcomes); (3) helping users to respond in more helpful ways (mechanisms); (4) addressing perceived and actual implementation factors (context); and (5) limited impact of user characteristics on DMHI outcomes (context).

**Conclusions:**

There is preliminary evidence that DMHIs can be delivered to people experiencing psychosis within trial settings, although use varied between individuals. Future implementation efforts may benefit from addressing contextual factors influencing DMHI use, including users’ treatment needs and preferences, everyday demands, and staff availability for blended interventions. Future research could evaluate implementation strategies, validate how and for whom DMHIs work, and embed process evaluation in trials.

**Trial Registration:**

PROSPERO CRD42024439117; https://www.crd.york.ac.uk/PROSPERO/view/CRD42024439117

## Introduction

Psychosis is characterized by strongly held unusual beliefs and often distressing sensory experiences, such as hearing voices [1], and can cause difficulties in many aspects of life, including interpersonal relationships, reduced quality of life, and increased risk of early death due to poorer health outcomes [2]. In the United Kingdom, clinical practice guidelines recommend psychological interventions, such as cognitive behavioral therapy or family interventions, for individuals with psychosis [1]. However, access to these therapies remains below recommended levels [3,4].

Given these persistent access challenges, attention has increasingly turned to digital mental health interventions (DMHIs), or technology-based treatment designed to improve users’ mental health [5], as a potential way to expand psychological support. Notably, a 2024 early value assessment by the National Institute for Health and Care Excellence in the United Kingdom highlighted DMHIs could be a promising treatment mode for those with psychosis [6]. Although evidence suggests DMHIs can reduce mental health symptoms experienced by those with psychosis [7,8], implementing these interventions in practice remains a persistent challenge [9-11].

To help bridge this DMHI evidence-practice gap, an international commission from Lancet Psychiatry in 2024 recommended adopting methods that embed implementation work throughout the research process [12]. One such research method is a process evaluation, or studies that are often embedded in randomized controlled trials (RCTs) that explore how, for whom, and where interventions work [13]. The most recent process evaluation guidance was published by the Medical Research Council (MRC) in the United Kingdom in 2015 [13]. Process evaluation in the MRC framework explores 3 outcomes (Textbox 1) [13].

**Medical Research Council process evaluation components [13].**

- Context: external factors that influence how an intervention is implemented or functions.
- Implementation: the quantity and quality of, and processes required for, intervention delivery.
- Mechanisms of impact: how an intervention produces an effect.

Some studies have used process evaluation to evaluate DMHIs used by those with psychosis [14-17]. Existing evidence highlights that digital treatments may work by empowering users or facilitating self-reflection [14-17], and has identified some barriers to implementation, including user-level (eg, competing life demands) [17] and service-level (eg, staff turnover) [16] factors. However, no review has synthesized process evaluation findings for DMHIs used by those with psychosis. Further, process evaluation is often underused [18] or inconsistently labeled as such [19,20], meaning relevant data may be difficult to identify. Consequently, the breadth, nature, and gaps in the available evidence base are currently unclear.

A scoping review is therefore warranted to understand what is currently known in this area and to identify key evidence gaps to inform future research, evaluation, and implementation efforts. In line with methodological recommendations [21,22], our review focuses on research conducted in the United Kingdom, as differences in health care systems and psychosis care pathways can limit comparisons between countries. Our research question was, “What is currently known about the context, implementation, and mechanisms of impact from RCTs predominantly conducted in the United Kingdom used by people with psychosis?”

## Methods

### Design

This review followed the Joanna Briggs Institute methodology for scoping reviews [23] and is reported in accordance with the PRISMA-ScR (Preferred Reporting Items for Systematic Reviews and Meta-Analyses extension for Scoping Reviews) [24] and Enhancing Transparency in the Reporting of Syntheses of Qualitative evidence [25] checklists (Tables S1 and S2 in Multimedia Appendix 1). The protocol was preregistered and can be accessed at PROSPERO (International Prospective Register of Systematic Reviews; CRD42024439117). No amendments were made to the protocol.

### Search Strategy and Eligibility Criteria

**Figure 1:**
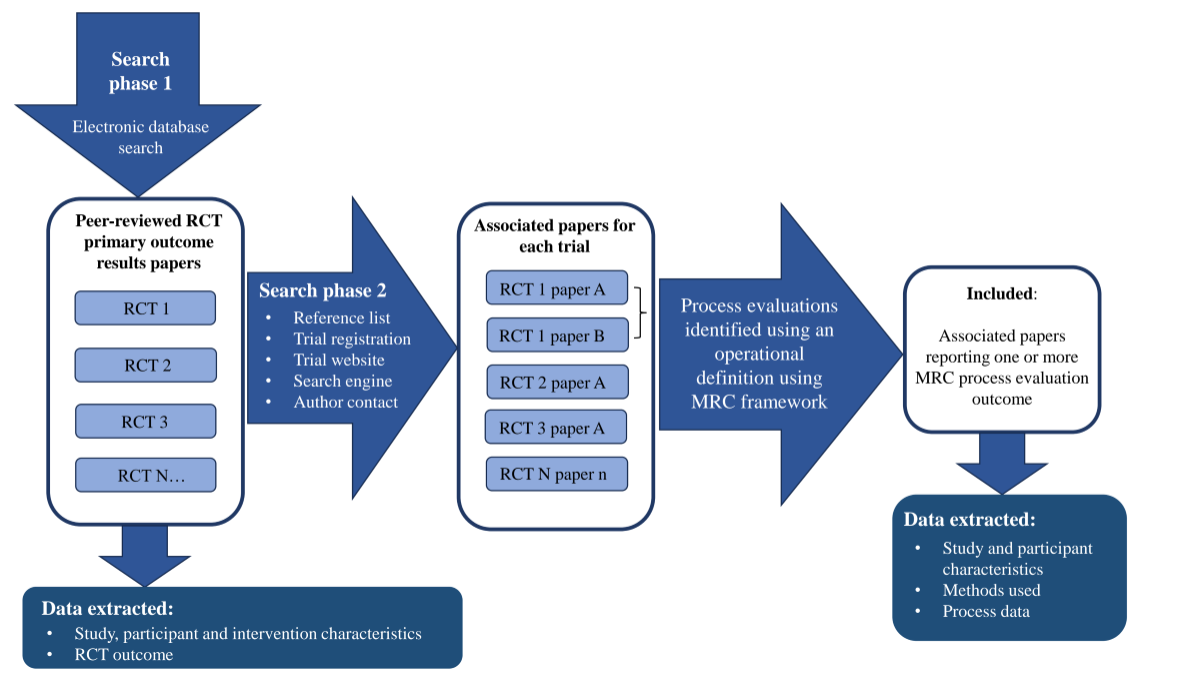
Method overview. MRC: Medical Research Council; RCT: randomized controlled trial.

A 2-phase search strategy, supported by Covidence software, modeled on a prior review of process evaluation [19], was used to first identify relevant RCTs, then associated papers (Figure 1 adapted from French et al [19]). This approach helped address inconsistent use of the term “process evaluation” [19,20] and is recommended for retrieving such studies [20].

Phase 1 identified DMHIs used by people with psychosis that were evaluated in an RCT (index RCTs) using preplanned database searches of PsycInfo and Cochrane Central Register of Controlled Trials on January 31, 2024, and October 22, 2025 (Table S1 in Multimedia Appendix 2). Studies included in phase 1 met the following requirements:

- Population: participants aged >14 years with psychosis established by either (1) a diagnosis of a psychotic disorder using a validated tool, or (2) a score on a standardized psychosis measure (indicating psychotic symptoms are the primary presenting difficulty), or (3) receipt of care from a specialist psychosis service.
- Intervention: digitally delivered, targeting a mental health difficulty.
- Studies: the main outcome paper (the first peer-reviewed paper publishing the prespecified primary outcome data) of peer-reviewed predominantly RCTs based in the United Kingdom (≥50% of samples in the United Kingdom in multicountry studies with comparable health care systems).

Phase 2 systematically identified peer-reviewed publications associated with index RCTs that reported process evaluation data. Following Cochrane guidance [26], process evaluations were identified using the 2015 MRC framework [13], irrespective of how the studies were labeled. Definitions and coding rules are provided in Table 1. Studies in phase 2 met the following requirements: reporting at least 1 process evaluation outcome from the 2015 MRC framework (context, implementation, or mechanisms of impact) [13] using qualitative, quantitative, or mixed methods, and being clearly linked to the main RCT (via trial registration or program grant).

In phases 1 and 2, masked reviewers (CH, SD, CF, and ALT) independently screened search results in Covidence using eligibility criteria on titles and abstracts, then the full text. Agreement between reviewers was calculated using Cohen κ for both screening stages in Covidence, and discrepancies in inclusion were resolved through discussion, with senior team members (PJ and CD) consulted where appropriate.

**Table 1:** Coding framework operationalizing process evaluation components [13].

Component and definition		Coding rules
**Context**		
	External factors that influence intervention implementation or functioning in a trial.	Code data about factors that are external to the intervention and its components. For intervention implementation, code data that influences how the intervention delivery or components are carried out. For intervention functioning, code data that influences the purpose or desired effect of interventions.
**Implementation**		
	How much and/or how well an intervention is used and/or delivered in a trial (implementation outcomes) [27].	For implementation outcomes, code data about any of the following: who participates in, uses, or delivers the intervention, how much the intervention is implemented, and the degree to which the intervention is implemented as intended.
	How delivery is achieved in a trial (implementation strategies) [28].	For implementation strategies, code data about the actions taken by researchers to enable or support delivery in a trial.
**Mechanisms of impact**		
	How interventions bring about change in a trial.	Code data about the intermediate structures between the intervention and the outcomes that produce an effect. Data can include intended and unintended mechanisms.

### Data Extraction and Critical Appraisal

Relevant data were electronically extracted in Covidence to reduce bias. Masked reviewers (CH, SD, and ALT) extracted summary characteristics from index RCTs (study, participant, and intervention characteristics and RCT outcome [positive/negative]) and process evaluation studies (study and participant characteristics and methods used). CH extracted process evaluation data using a predefined coding guide (Table 1). Missing data were not sought from the authors. The methodological quality of associated publications was not assessed because a validated tool for process evaluations has not been developed [20], and inclusion of all studies in qualitative syntheses is recommended [29]. Index RCTs were independently assessed by masked reviewers (CH and RT) using the Cochrane risk-of-bias 2 tool, evaluating five domains: (1) randomization procedures, (2) deviations from intended interventions, (3) missing outcome data, (4) measurement of the outcome, and (5) selection of the reported result [30]. As scoping reviews do not typically exclude studies based on methodological quality, these assessments were conducted to provide contextual information on the included evidence rather than to inform study inclusion.

### Data Synthesis

**Figure 2:**
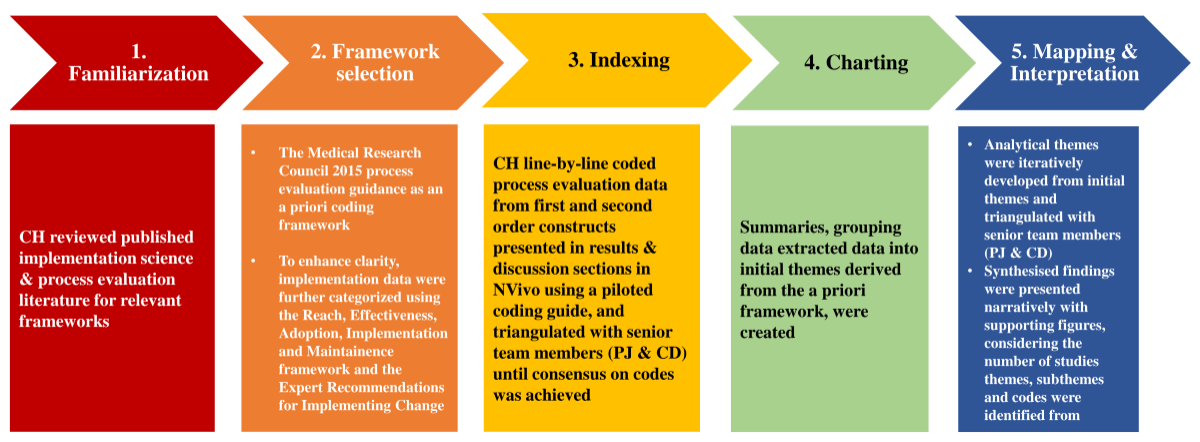
Framework synthesis overview.

We used a constructivist approach [31] with a critical realist perspective [32]. A convergent data-based synthesis design, supported by NVivo (Lumivero), was used to qualitatively synthesize process evaluation data deductively [33]. Framework synthesis, using the 2015 MRC process evaluation framework [13], guided analysis in five stages: (1) familiarization, (2) framework selection, (3) indexing, (4) charting, (5) mapping and interpretation (Figure 2) [34]. Process evaluation data were indexed using the coding framework outlined in Table 1. To enhance the interpretability of findings, data coded within the implementation domain in the MRC framework were categorized into implementation outcomes using relevant domains from the Reach, Effectiveness, Adoption, Implementation, and Maintenance framework, which includes 5 outcomes relevant to evaluating implementation [27]. Data were also coded as implementation strategies (actions taken to support delivery) from the Expert Recommendations for Implementing Change (ERIC), which includes 73 defined implementation strategies that have been ranked by implementation experts [28]. These are established implementation science frameworks that are commonly used to structure and evaluate implementation processes and outcomes in health care research [35,36], and, therefore, organized data using terminology consistent with the implementation science field. CH was the primary analyst, iterated stages as necessary, and triangulated findings with senior researchers (PJ and CD; Figure 2). Results are presented as a narrative synthesis. To enhance synthesis clarity, we applied a frequency criterion adapted from Graham-Rowe and colleagues [37], whereby the number of studies associated with themes was considered in the narrative synthesis. The coding tree is reported in Multimedia Appendix 2.

### Reflexivity Statement

The authors have experience in intervention delivery and implementation and DMHI projects. The team includes a PhD candidate completing a doctorate on the implementation of a DMHI for paranoia (CH), clinical psychologists with experience delivering interventions to service users experiencing psychosis (PJ and ALT), an expert in psychosis research who has conceptualized a DMHI for paranoia included in our review (CBM-pa; JY) [38], researchers involved in evaluating an adapted version of this DMHI in an RCT (CH, SD, CF, ALT, and RT) [39], and a senior researcher with experience implementing health interventions (CD). We recognize that our professional and research backgrounds and interest in DMHI implementation may have influenced analyses. Our combined clinical and research experience may have provided a nuanced understanding of RCT implementation processes and mechanisms of impact. Direct engagement with DMHI participants provided insight into end user perspectives, which may have informed attention to the relational and practical aspects of implementation. Involvement of senior authors not directly engaged with DMHI users (PJ, CD, and JY) provided an external perspective that may have counterbalanced potential biases. Bias was also mitigated in quality assessment and interpretation, as JY was not involved in the quality rating of CBM-pa or the analysis of intervention findings.

## Results

### Overview

**Figure 3:**
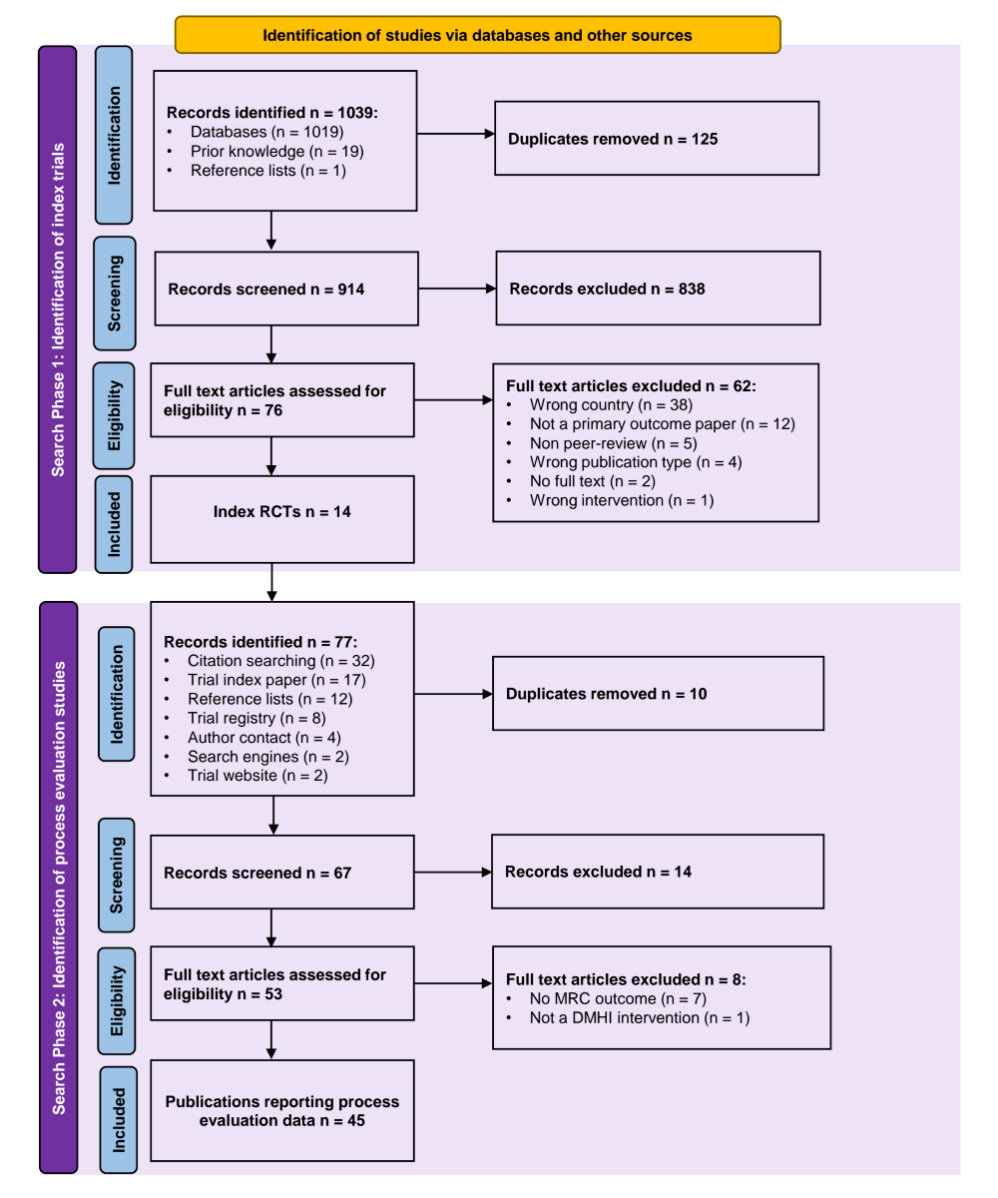
Search flow diagram. DMHI: digital mental health intervention; MRC: Medical Research Council; RCT: randomized controlled trial.

The search results are summarized in Figure 3. Phase 1 initially identified 1039 papers, 914 of which were excluded after deduplication, primarily because they were protocol papers, were conducted outside of the United Kingdom, or were not primary outcome papers. In phase 1, a total of 14 index RCTs were included. Agreement in phase 1 was moderate to substantial for title/abstract screening (k=0.55-0.72), and substantial to perfect (k=0.62-1) at full text screening. Phase 2 screened 77 papers for process evaluation data (14 were index RCTs included in phase 1, and 63 papers were systematically identified in phase 2). From this, 10 duplicates and 22 papers (mostly protocol papers and papers not reporting process evaluation data) were excluded. In phase 2, a total of 45 papers reporting process evaluation data were included (13 index RCTs and 32 associated papers). Agreement in phase 2 was perfect for title/abstract screening (k=0.87-1) and substantial at full text (k=0.75).

### Index RCT Methodological Quality

Quality ratings of index RCTs are included in Table S2 in Multimedia Appendix 2. Most disagreements arose in domain 2 (deviations from the intended interventions) and were resolved by discussion between raters (CH and RT). Half (7/14, 50%) RCTs showed some risk of bias, primarily due to bias arising from deviations from intended interventions or outcome measurement [40-44]. The remaining 7 RCTs had low risk of bias [38,45-47].

### Index RCT Summary Characteristics

Summary characteristics of index RCTs are reported in full in Table S3 in Multimedia Appendix 2, and intervention characteristics are presented in Table 2. Most DMHIs targeted psychosis specific symptoms (12/14, 86%) and were delivered in a participant-chosen or any setting (9/14, 64%). Many (8/14, 57%) RCTs were conducted from a London-based university [38,44-47] or the University of Oxford [40-42]. One RCT was conducted outside of England in Scotland and Australia, but was included because less than half of the participants were Australian (24/73, 29%) [48]. Based on available data, intervention users were mostly White (656/1023, 64%), male (653/1023, 64%), had a mean age of 38.6 (SD 1.5) years, and a primary diagnosis of a psychotic disorder (890/1023, 87%).

**Table 2:** Intervention characteristics (N=14).

Intervention	Index RCT^a^ reference	Intervention target		Delivery setting				Delivery mode		
		Psychosis specific symptom	General mental health	Any setting/participant choice	Clinical setting/study site	Not stated	Virtual reality		Computer/web	Smartphone
Actissist	Bucci et al (2018) [43]	✔		✔						✔
Actissist 2.0	Bucci et al (2024) [49]	✔		✔						✔
AVATAR	Craig et al (2018) [45]	✔			✔				✔	
AVATAR2	Garety et al 2024 [50]	✔		✔	✔				✔	
CBM-pa	Yiend et al (2023) [38]		✔		✔				✔	
EMPOWER	Gumley et al (2022) [48]	✔		✔						✔
—^b^	Freeman et al (2016) [40]	✔			✔		✔			
gameChange	Freeman et al (2022) [41]		✔	✔			✔			
—	Leff et al (2013) [44]	✔				✔			✔	
MUSE	Dodgson et al (2025) [51]	✔		✔					✔	
MUSE-FEP	Dudley et al (2024) [52]	✔		✔					✔	
SloMo	Garety et al (2021) [47]	✔		✔					✔	✔
THRIVE	Freeman et al (2023) [42]	✔		✔			✔			
V-NeST	Cella et al (2022) [46]	✔				✔	✔			

^a^RCT: randomized controlled trial.

^b^None given.

### Studies Reporting Process Data Summary Characteristics

Summary characteristics of studies reporting process evaluation data are reported in full in Table S4 in Multimedia Appendix 2 and summarized in Table 3. Most studies were published between 2021 and 2023 (24/45, 53%), quantitative (19/45, 42%), used trial data (31/45, 68%), evaluated the study intervention (39/45, 87%), and did not describe the project as a process evaluation (43/45, 96%).

**Table 3:** Characteristics of studies reporting process evaluation data (N=45).

Process evaluation characteristics		Value, n (%)^a^
**Publication year**		
	2013-2016	2 (4)
	2017-2020	11 (24)
	2021-2023	24 (53)
	2024-2025	8 (18)
**Study design**		
	Quantitative	19 (42)
	Qualitative	16 (35)
	Mixed methods	10 (22)
**Process scope**		
	Study intervention	39 (87)
	Intervention implementation	9 (20)
	Trial procedures	9 (20)
**Process evaluation data source**		
	Trial data	31 (68)
	Additional stakeholder feedback	18 (40)
	Author description	8 (18)
**Labeled as process evaluation**		
	Yes	2 (4)
	No	43 (96)

^a^Some papers fall into more than one category, so categories may add to more than the overall total (N=45).

There was a range of participants and contributors to studies reporting process evaluation data (Table S4 in Multimedia Appendix 2), with trial participants comprising the majority (1856/2155, 86%) across 37 (82%) studies. Other contributors included health care professionals (195/2155, 9%) and individuals with lived experience (184/2155, 9%). However, missing sociodemographic data in 17 (37%) studies limited the ability to accurately summarize this information.

### Framework Synthesis Findings

#### Overview

**Figure 4:**
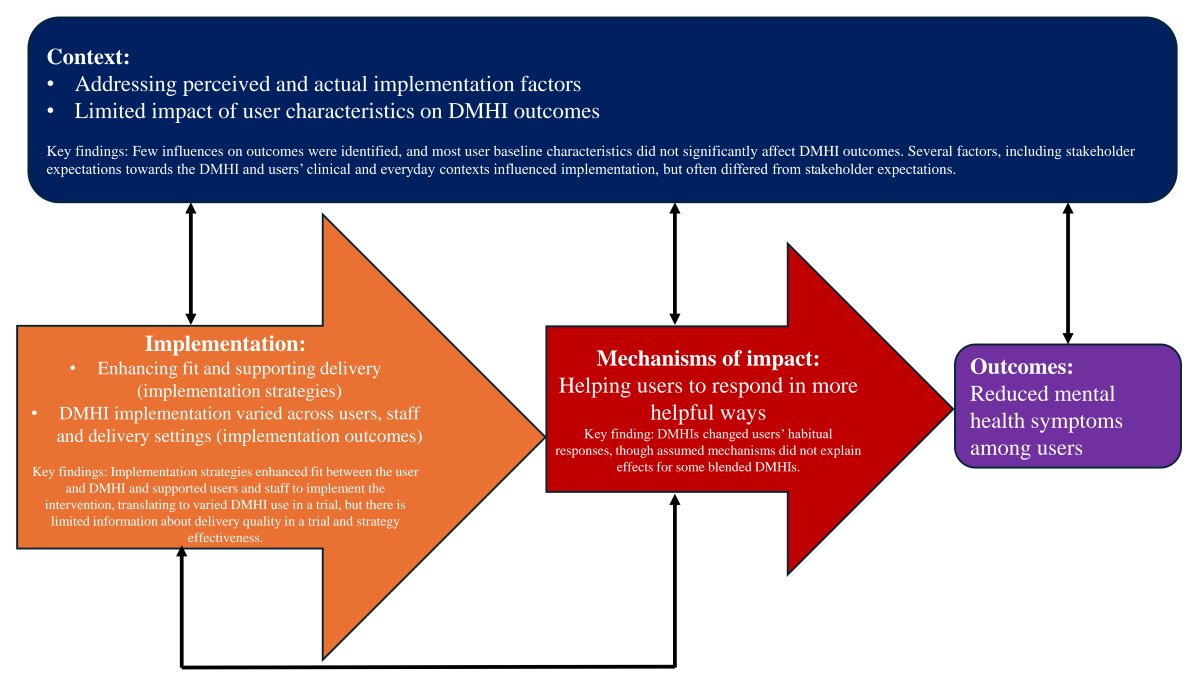
Framework synthesis themes summarized using the Medical Research Council process evaluation diagram. DMHI: digital mental health intervention.

Framework synthesis of process evaluation data generated 5 themes (Figure 4 [13]), all of which were coded within the components deductively derived from the MRC framework (context, implementation, and mechanisms of impact) [13].

#### Implementation

##### Overview

Most findings related to implementation, defined in this review as (1) the actions taken by trial teams to support DMHI delivery in an RCT (implementation strategies) [28] and (2) how much and by whom, a DMHI was used and delivered within an RCT, including if an intervention was delivered as planned (implementation outcomes) [27] (38/45, 84% studies; 13/13, 100% DMHIs). Two themes, mapped to the MRC framework, were created: (1) enhancing fit and supporting delivery (implementation strategies) and (2) DMHI implementation varied across users, staff, and delivery settings (implementation outcomes).

##### Enhancing Fit and Supporting Delivery (Implementation Strategies)

Implementation strategies were identified in 34 (76%) studies across all 13 (100%) DMHIs. These data, identified from stakeholder feedback, trial data, and author interpretation, were summarized in two subthemes: (1) strategies to improve fit and (2) strategies to support use and delivery.

#### Strategies to Improve Fit

Most trial teams (12/13, 92%) used implementation strategies that aimed to improve the fit between the DMHI and the user. This included the following implementation strategies from the ERIC: adapt and tailor to context, engage consumers, and change infrastructure [28]. Most trial teams (10/12, 83%) designed DMHIs that could be adapted to the user. This included allowing users to personalize virtual reality– (VR), web-, or app-based intervention content in 7 (58%) DMHIs [41,43,49,52-61], (2) offering on-demand access to 4 (33%) interventions, primarily via apps [43,44,49,53,55,59-62], and enabling multisetting delivery for 3 (25%) blended DMHIs [41,42,49,63]. These features could improve fit with users’ needs, schedules, or delivery preferences. Stakeholders consistently valued DMHI flexibility [43,55,58,61] or requested it when lacking [17,62,64]. Half of the trial teams (6/12, 50%) also involved patients and potential consumers in the implementation effort, using co-design or user-centered design methods intended to make the DMHI more relevant to end users [49,60,64-69]. Three of these trial teams (Actissist 2.0, AVATAR2, and SloMo) attributed engagement to these approaches [47,49,55,69,70]. Finally, 3 RCTs (Actissist, Actissist 2.0, and MUSE) addressed potential infrastructural barriers by enabling access to the technology needed for the DMHI. This included delivering a blended DMHI using existing National Health Service (NHS) technology [71] and providing study phones, which were used by 70% [49] and 87% [43] of participants.

#### Strategies to Support Use and Delivery

Trial teams (11/12, 92%) also used implementation strategies that enhanced DMHI delivery and use by assisting intervention users and staff responsible for delivering the intervention. This included the following implementation strategies from the ERIC: training and educating stakeholders and providing interactive assistance [28]. Most strategies focused on supporting users in the RCT. Many (9/12, 75%) teams reported providing interactive assistance to users, primarily via staff involvement, though 2 DMHIs (SloMo and gameChange) also incorporated digitized human support [55,58,65]. In 2 DMHIs (CBM-pa and Actissist), trained researchers provided professional support specifically for the trial [53,64]. Across DMHIs, staff offered practical and technical assistance to facilitate DMHI use [17,46,53,58-60,62,65,72-74]. For blended DMHIs, users frequently highlighted the importance of positive relationships with the delivery staff. Positive relationships were developed through delivery staff’s nonspecific therapeutic skills, including active listening, empathy [17,55,58,60,62,73], and encouragement [58,62,73]. User feedback on interpersonal support was mixed: some users described assistance as central to engaging with the DMHI [58,62,73], while others viewed it as less important or preferred using the intervention independently [46,58,60,61,64]. No clear patterns of user preference emerged according to intervention features. Fewer trial teams reported implementation strategies aimed at supporting delivery staff. A total of 4 (33%) teams reported training and educating delivery staff on the DMHI prior to the RCT [51,55,65,75].

##### DMHI Implementation Varied Across Users, Staff, and Delivery Settings (Implementation Outcomes)

Implementation outcomes data were identified in 21 (47%) studies across all 13 (100%) DMHIs. Most implementation outcome data focused on individual-level implementation, particularly intervention reach and DMHI use, whereas less information was available about implementation by delivery staff or services.

User-level implementation outcomes are primarily related to intervention reach (the extent to which intended users participated in the intervention) and DMHI use within RCTs (13/13, 100% DMHIs). Reach was often operationalized as participation rates and reported by all but 1 team [49]. Across studies, the proportion of eligible individuals who participated in trials varied substantially, ranging from 27% [46] to 100% [38,41,42,45]. No clear patterns emerged regarding variation in participation rates according to DMHI characteristics or trial team.

Information about who was considered suitable to participate in DMHI trials was often incompletely reported. Several teams stated that participants were excluded because they did not meet eligibility criteria, without specifying which criteria were unmet [43,46,47,52]. Where reasons for exclusion were reported, data most commonly indicated participants were those who were currently experiencing the mental health difficulty targeted by the DMHI [41,42,45], and not receiving concurrent psychological therapy [38,41,45,52] or considered high risk, such as being acutely unwell, hospitalized, or experiencing suicidal intent [42,45]. Just 4 (31%) trials teams reported whether participants were representative of the intended target population, including those experiencing psychosis [38,47,50] or local service populations [51]. Where discussed, representativeness was primarily assessed using basic demographic characteristics. Trial samples were commonly described as predominantly White, male, or unemployed, with these characteristics used to justify representativeness compared to target populations [38,47,50,51].

Quantitative trial data indicated that DMHI use varied among users of both blended (DMHIs are delivered in a session and guided by a member of staff, 9/13, 69%) and self-administered (DMHIs are used independently, 4/13, 31%) delivery models. One intervention (SloMo) used both models, allowing in-session and independent DMHI use [47]. Session attendance for blended DMHIs ranged from 0% to 100% [41,42,45,46,50-52]. For self-administered DMHIs, use was operationalized using adherence criteria, with the proportion of users meeting this ranging between 65.4% [63] and 91% [48]. Two RCTs (Actissist and Actissist 2.0) reported detailed usage data showing that app engagement declined by 67%-67.5% over 12-week intervention periods, with users accessing the app at varied times of day [53,54].

Implementation outcomes relating to DMHI delivery staff and services were less frequently reported (9/13, 69% trial teams). A total of 8 (62%) trial teams reported the adoption (implementation by settings or delivery staff) of DMHIs in an RCT. However, these studies rarely reported formal adoption metrics, such as the proportion of settings or clinicians willing to deliver the DMHI. Instead, adoption was typically inferred indirectly from descriptions of delivery staff and their level of expertise (7/8, 88% trial teams). Across these studies, the representativeness of delivery staff appeared variable, although reporting was generally limited to professional role descriptions. Overall, 4 (58%) blended DMHIs were delivered by therapists who often had expertise in psychological therapies for psychosis [44,45,55]. In contrast, 3 (42%) DMHIs were delivered by staff without specialist psychotherapy roles [51,52,65], such as assistant psychologists or care coordinators. Further, a few trial teams reported how successfully delivery staff implemented the DMHI in an RCT. Only 3 (23%) of these teams assessed therapist adherence and skill in delivering AVATAR1 and MUSE therapies, reporting high fidelity to therapy manuals [45,51,52]. Another trial (CBM-pa) noted successful delivery without supporting data [38]. Finally, only 2 trial teams reported adoption at the service level. EMPOWER recruited and retained all 8 participating community mental health services throughout the trial [48], whereas the MUSE-FEP reported substantial site attrition, with 7 of 15 recruiting sites withdrawing during the trial due to the COVID-19 pandemic [52].

#### Context

##### Overview

All interventions reported contextual findings, or external factors that influenced DMHI implementation or functioning in RCTs (33/45, 73% studies; 13/13, 100% DMHIs). Contextual findings were explored quantitatively using moderation analyses (10/33, 30% studies) [41,42,47,48,50,53,54,63,76,77], and qualitatively using stakeholder consultation (11/33, 33% studies) [16,17,46,58,60,61,64,73,78,79], and reasons for trial participation or withdrawal (12/33, 36% studies) [38,43,45,46,49-52,55,58,75,80]. Two themes that aligned with the MRC framework components were created: (1) addressing perceived and actual implementation factors and (2) the limited impact of user characteristics on DMHI outcomes [13].

##### Addressing Perceived and Actual Implementation Factors

###### Overview

Contextual data about implementation (factors that influenced how a DMHI was delivered in RCTs) were identified in 26 (58%) studies across 12 (92%) DMHIs. Three subthemes were created from stakeholder feedback [16,17,46,55,57,58,60-62,64,73,78,79], trial data [43-46,48,50,52-55,63,71,75,80], and author description [59]: (1) stakeholder misconceptions about user-related barriers, (2) service-level barriers: perceptions and realities, and (3) hope and help: positive expectations facilitate implementation.

###### Stakeholder Misconceptions About User-Related Barriers

Health care professionals and some service users held generalized concerns that psychosis-related and technology-related factors would hinder DMHI implementation. Staff in pre–COVID-19 studies anticipated that users’ limited technology access and digital skills would act as key implementation barriers [78,79]. However, process evaluation data contradicted these assumptions: users engaged with a range of DMHI modalities, including computer-based, smartphone-based, and VR-based interventions, regardless of digital confidence [17,46,55,60,64]. Only SloMo reported higher engagement among more technologically confident users before the RCT [63]. Concerns about users’ technology-related paranoia or illness severity were also overgeneralized [57,78,79] as not all users had experienced technology-related paranoia [62,73], and some used DMHIs despite concerns [60,67,73].

Actual implementation barriers included different psychosis symptoms (auditory hallucinations [44,59,62] and negative symptoms [46]), everyday life and situational factors outside of therapy (eg, users having other time commitments such as working or studying [17,43,53,73]), and DMHIs not meeting users’ treatment needs or preferences (eg, lack of interest in the DMHI or RCT [41-43,45-48,51,52,70] or feeling that the DMHI was not suited to them [17,43,45,55,57,75]). Demographic factors had a limited influence on use in 3 (25%) DMHIs: older age [53,54], White ethnicity [54], and higher baseline smartphone confidence [63] were associated with higher engagement. A number of other factors were generally nonsignificant (gender, education, employment, and prior smartphone ownership) [53,54,63].

###### Service-Level Barriers: Perceptions and Realities

Stakeholders (service users and staff) often perceived psychosis-specialist services as underresourced for DMHI delivery, citing staff availability, physical space, and technology constraints [57,78,79]. These concerns were particularly noted for interventions requiring clinical involvement (EMPOWER and gameChange) [78,79], the latter with VR-specific equipment and spatial needs [45]. Evidence from 2 RCTs (EMPOWER and MUSE) indicated staff resource constraints linked to high workloads and turnover hindered recruitment [16], staff training [51], and data collection [48]. In contrast, staff perceptions for Actissist did not align with its delivery model [43,78], as the smartphone-based intervention required neither the assumed NHS systems nor staff support for delivery [43].

###### Hope and Help: Positive Expectations Facilitate Implementation

Positive expectations among users and health care professionals facilitated DMHI use. Most users tried the DMHI expecting personal benefit from use [17,58,60,62,73,79,80], while some users were motivated by helping others [62,80] or trying something different within their treatment [46,62]. Staff reasons for wanting to deliver a DMHI, though less frequently reported, aligned with user expectations as some anticipated benefits for their clients [57,78].

##### Limited Impact of User Characteristics on DMHI Outcomes

A few contextual factors influenced DMHI functioning. Only 2 (15%) DMHIs (gameChange and MUSE) identified 3 moderators, reporting enhanced effects among participants with higher baseline illness severity [76], earlier psychosis symptom onset [50], and those who felt valued from using the intervention [77]. Across 5 (38%) DMHIs, most user baseline characteristics did not affect outcomes; no consistent patterns emerged, though gender was not found to moderate effects [41,42,47,50,52].

#### Mechanisms of Impact: Helping Users to Respond in More Helpful Ways

Mechanisms of impact data, or how DMHIs produced change in an RCT, were identified in most (10/13, 77%) DMHIs across 19 (42%) studies. Evidence predominantly came from qualitative sources, including DMHI user feedback (8/19, 42% studies [17,43,58,60-62,64,73]), intervention content (4/19, 21% studies [55,72,74,75]), and author commentary (2/19, 10% studies [51,59]), while only 4 studies quantitatively tested hypothesized mechanisms [38,41,42,47].

Across the 10 (77%) interventions, DMHIs appeared to help users respond to difficult thoughts or situations in more helpful ways by changing their habitual thinking (eg, belief inflexibility, beliefs about voices, or interpretation biases [17,38,41,45,47,55,60,62,64,72,75]) or behaviors (eg, reducing defense behaviors or increasing assertive behaviors [41,42,45,55,58,59,61,72-74]). Many (7/13, 54%) interventions were theoretically informed and targeted putative maintaining mechanisms [17,38,41-43,47,49]. Changes were achieved by replacing unhelpful responses (eg, negative interpretations of everyday situations or avoidance of everyday social situations that were perceived to be unsafe) with more helpful responses (eg, seeking alternative, less personally threatening explanations [38,47,55,58,60,62,64], or entering challenging situations [41,58]). For some users, changing habitual responses was achieved by becoming aware they were responding in unhelpful ways [55,58,60,61,64], understanding why they responded in unhelpful ways [55,72,75], or practicing responding in new ways [55,58,60,62].

However, assumed mechanisms did not consistently explain outcomes in 4 DMHIs with a blended delivery model delivered via an app [47,48] or VR [41,42]. In 3 of these DMHIs, targeted mechanisms (safety beliefs [41,42], defense behaviors [42], or a reasoning bias [47]) did not mediate intervention effects, even when symptom improvement occurred [41,47]. In another DMHI, EMPOWER, user interviews revealed that not all users experienced changes to the targeted mechanism (relapse appraisal) [17].

## Discussion

### Principal Findings

#### Overview

We created 5 themes after synthesizing process evaluation data from 45 peer-reviewed papers evaluating 13 DMHIs used by those experiencing psychosis in RCTs predominantly based in the United Kingdom. These themes aligned with deductive MRC framework domains: implementation, context, and mechanisms of impact [13].

#### Implementation

Overall, implementation findings indicated that research in trials primarily focused on the implementation at the level of the individual user, with substantially less information available regarding delivery staff and services. Current DMHI implementation research for psychosis has therefore prioritized understanding user engagement, while providing limited insight into whether services can realistically deliver DMHIs.

Implementation data suggested it may be feasible to deliver DMHIs to people experiencing psychosis in a trial. Across studies, users engaged with DMHIs in a trial setting. Where considered, trial participants were often described as broadly representative of populations with psychosis or local services [38,47,50,71], suggesting that DMHI delivery may be feasible among groups currently receiving psychosis care. However, representativeness was inconsistently assessed and typically based on basic demographic characteristics. Further, while DMHI use in a trial varied between individuals, this finding is consistent with previous reviews [10,81,82] and attendance patterns observed in psychological therapies for psychosis more broadly [83]. Consistency in variation may suggest that some level of disengagement could be expected when implementing DMHIs and, importantly, may not indicate implementation failure unique or issues inherent in this delivery mode. Variation in use persisted across blended and self-delivered DMHI using different technology, suggesting that DMHI use may be shaped by external, nontherapy related factors, including fluctuating needs, user autonomy in care, or unmet treatment preferences, all factors identified in this (see the “Context” section below) and other work [84].

Implementation strategies similarly primarily focused on addressing potential barriers at the user level, including intervention accessibility, usability, and user support needs. Trial teams often aimed to improve the fit between the DMHI and the user through intervention flexibility, stakeholder involvement, tailored support, and improving access to necessary technology. These approaches align with broader recommendations both in the DMHI [11,85] and implementation science [28] literature. Consequently, although implementation science frameworks were rarely explicitly used, trial teams were often already undertaking implementation-focused work. However, greater use of implementation science frameworks may help structure, evaluate, and consistently report these efforts and enable a better understanding of how DMHIs can be successfully implemented. Stakeholder feedback highlighted some of these strategies, including ensuring the intervention content, delivery approach, and amount of professional support provided [43,55,58,61], as potential design priorities for future work. However, despite routine use of promising implementation strategies, no studies evaluated whether these strategies improved implementation outcomes, and variation in DMHI use persisted across interventions. Further, despite DMHIs often being discussed as scalable or resource-efficient interventions [86], implementation costs were not reported in the included studies. Our review challenges assumptions that DMHIs are inherently low-resource interventions because many implementation strategies likely require substantial resources, including money (eg, payment for specialist workers and end users in co-design processes) and time (eg, developing and delivering training). Future work clarifying these “hidden” costs, in addition to evaluating implementation strategy effectiveness, would better establish how best to implement DMHIs for psychosis.

In contrast, evidence relating to delivery staff and services remained limited across implementation strategies and outcomes. Available evidence primarily focused on the delivery staff, with less attention given to wider service or organizational implementation requirements. Although all included DMHIs included professional support in delivery, only 3 (23%) trial teams evaluated delivery staff competency [45,51,52], despite staff being important determinants of successful delivery [10]. Few studies reported how staff were trained to deliver DMHIs, limiting understanding of the workforce support required to deliver these interventions consistently and to a high standard. However, blended DMHIs appeared deliverable by staff with differing levels of expertise, ranging from highly specialized therapists to broader mental health workforces in the United Kingdom. This suggests that workforce requirements and implementation demands may differ depending on the specific DMHI. Beyond delivery staff, implementation within health care services is also shaped by organizational factors, including available technology, delivery space, and financial resources [10]. These factors were rarely evaluated in included studies, limiting understanding of what resources are needed to implement DMHIs both in and after trials.

#### Context

Most studies (33/45, 73%) reported contextual findings, primarily relating to DMHI implementation, whereas few examined the influence of context on DMHI outcomes. Only 2 (15%) DMHIs identified moderators, including some illness factors and feeling valued from DMHI use [50,76]. Across 5 (38%) DMHIs, most user baseline characteristics did not significantly affect treatment outcomes [41,42,47,50,52]. While this could suggest that included DMHIs may work broadly across different user profiles, conclusions about subgroup differences remain tentative due to the small number of studies examining moderators and variability in examined moderators. Notably, the absence of gender moderation across several DMHIs contrasts with previous reviews [10,82], potentially reflecting differences in intervention type or populations. These inconsistencies highlight the need for more nuanced research to determine for whom DMHIs are most effective.

A key finding was the role of stakeholder DMHI appraisals. First, appraisals of the intervention shaped implementation, including stakeholder appraisals about DMHI relevance, fit with treatment needs, and expected benefits [20,48,52,64]. This emphasizes the importance of supporting user choice and aligning DMHIs with individual preferences (see the “Implementation” section above). Second, stakeholder misconceptions about user- and service-related barriers were common. Pretrial assumptions often overestimated difficulties due to users’ limited digital literacy, device availability, or technology-related paranoia [78,79], yet process evaluation data showed users engaged with a range of DMHI modalities regardless of prior digital experience [17,46,55,60,64], and concerns regarding technology-related paranoia were overgeneralized [62,73]. Only 1 intervention (SloMo) demonstrated higher engagement among more digitally confident users [63], suggesting that technological familiarity may enhance, but is not essential for, use. Service-level constraints were also highlighted. Service staff often cited limited resources as barriers [78], though evidence linking resource limitations to implementation was limited but highlighted staff availability as a relevant factor [16,48]. Further, staff perceptions did not always match actual intervention demands [78]. Given staff are important determinants of DMHI implementation [11,87], addressing misconceptions about DMHI users and delivery requirements may be critical. However, most studies predated COVID-19 [78,79], meaning attitudes toward DMHIs and user capability may have changed after the pandemic forced a move to almost entirely remote services in the United Kingdom due to 2 years of prolonged lockdowns [88].

User-reported barriers differed from assumed factors, including other psychosis-related symptoms (auditory hallucinations [59,62] and negative symptoms [46]) and life commitments outside of therapy [17,53]. These findings underscore the importance of addressing real, not assumed, contextual factors; as DMHI use is shaped by both clinical and everyday contexts, this may further reinforce the potential value of flexible intervention design to optimize use (see the “Implementation” section above).

#### Mechanisms of Impact

Evidence for DMHI mechanisms remains limited. Process evaluation data suggested DMHIs reduced users’ mental health symptoms by supporting more adaptive cognitive and behavioral responses to challenging situations. This mechanism was observed across different intervention modes and targets, though the specific mechanisms varied and were often not clearly delineated as such, which impeded review syntheses. However, evidence for these mechanisms is tentative and is currently insufficiently tested and validated. Most insights were derived from qualitative sources (user feedback or intervention content), rather than recommended quantitative hypothesis-driven approaches [89], which were reported in only 4 (10%) studies [38,41,42,47]. This limited the ability to confirm whether these processes causally explained symptom change. Further, hypothesized mechanisms were not consistently activated. Some blended DMHIs demonstrated effects through assumed mechanisms, such as belief inflexibility or defense behaviors [38,64], while others reported symptom improvements that were not mediated by hypothesized mechanisms (safety beliefs, reasoning, and safety behaviors) [41,42,47], suggesting that DMHIs may work through alternative or nonspecific processes. For interventions delivered via blended delivery models, intrapersonal factors, such as a therapeutic alliance, which has been identified as a driver of change when treating psychosis [90], may play an unrecognized role in driving change. Further, variability in mechanistic responses, such as inconsistent changes to reduced fear of relapse among EMPOWER users [17], highlights the likelihood that mechanisms function differently across users and contexts (see the “Context” section above).

### Comparison to Prior Work

Unlike previous reviews focusing on the effectiveness of DMHIs for psychosis [7,8,91], this synthesis of process evaluation data extends the understanding of implementation science in this area. Further, our review extends prior syntheses of process evaluation in other health domains, such as physical health interventions [18,92]. Consistent with broader findings that process evaluation remains underused across research fields [18,92] and that implementation science remains underapplied in DMHI research [93], we found limited use of process evaluation, with only 1 trial explicitly adopting this approach [16,17]. Although most DMHI research remains service-based [9,10], our findings show that both user-related and service-related factors can shape engagement and outcomes, and may provide a starting point for identifying priorities for future research and implementation efforts in and outside of services.

### Future Directions

**Figure 5:**
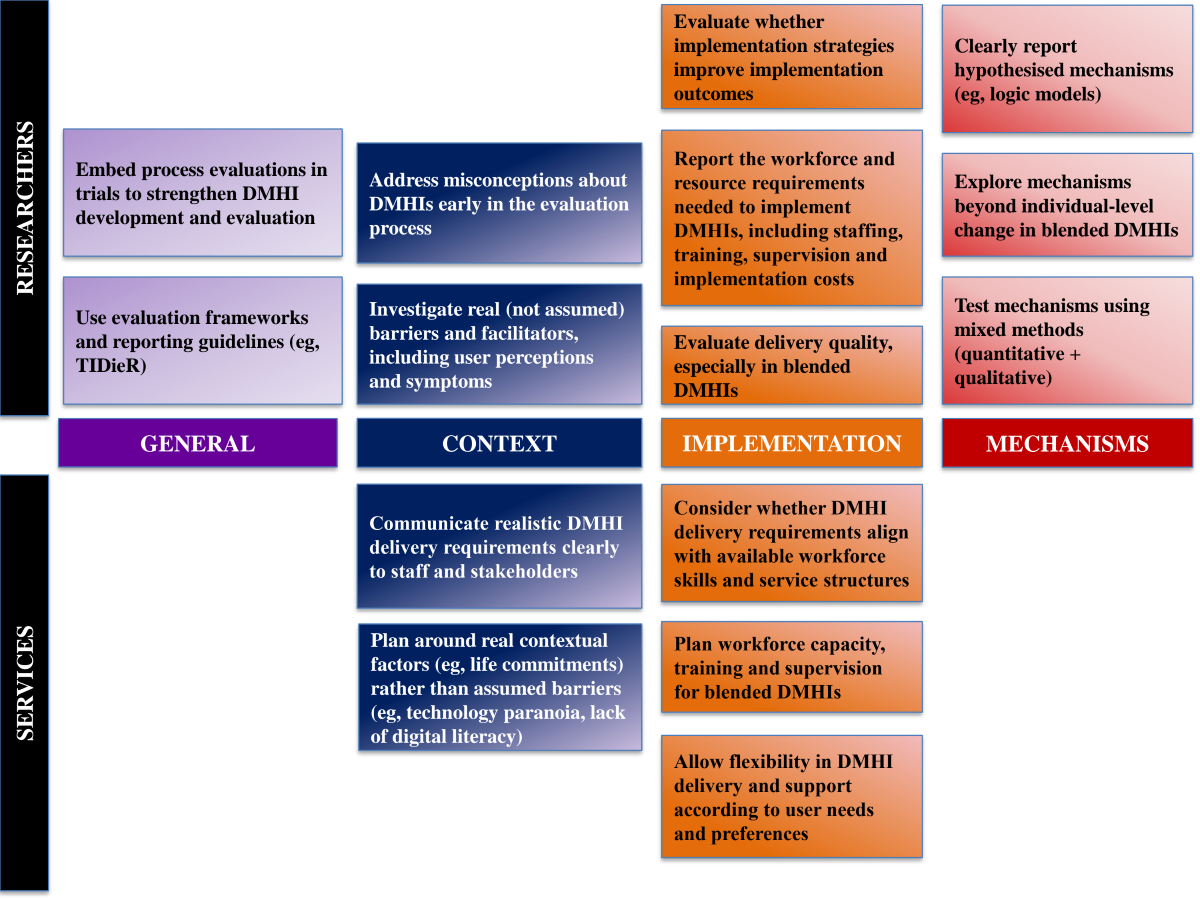
Recommendations for researchers and services. DMHI: digital mental health intervention; TIDieR: Template for Intervention Description and Replication.

Our review highlights several priorities to strengthen DMHI evaluation and implementation (Figure 5). For researchers, key steps include embedding process evaluation in trials using established frameworks and reporting guidelines, further clarification of how DMHIs reduce user mental health symptoms, and routine assessment of the delivery quality of blended DMHIs. Future research could evaluate which implementation strategies improve implementation outcomes. Further work is needed to clarify the workforce, organizational, and resource requirements needed to deliver DMHIs consistently, including staff training, supervision, and implementation costs. For services, priorities include clearly communicating DMHI requirements to staff, addressing real, not assumed, contextual factors, and adopting flexible delivery approaches.

### Review Strengths and Limitations

This review has several strengths. To our knowledge, it is the first review to synthesize process evaluation data in this field. Broad inclusion criteria, aligned with relevant guidance [20], enabled us to capture relevant studies irrespective of authors’ use of the term “process evaluation.” The lack of date and method restrictions and updated searches ensured comprehensive and up-to-date coverage. Rigorous methods, including review preregistration, double, masked screening, and data extraction and use of an established process evaluation framework for synthesis, helped minimize bias. Finally, unlike other scoping reviews [94], our use of established implementation science frameworks [13,27,28] helped ensure our findings were appropriately documented, which may help inform the broader field.

Several limitations should be noted. As in another review [92], most studies did not explicitly use process evaluation terminology, and data were primarily synthesized by a single author (CH), which may have introduced subjectivity despite the use of an established framework. The framework was operationalized at the theme level to enhance usability, possibly limiting analytical depth. Use of the frequency-based criterion from Graham-Rowe and colleagues [37] in the narrative synthesis may underemphasize less commonly reported findings. Extracting process evaluation data from only the results and discussion sections may have excluded relevant information from the methods sections. Many trial teams (10/13, 77%) described implementation processes (eg, staff training and device provision) in methods sections without supporting data, which were not captured in our syntheses. Finally, 1 included DMHI was implemented in the United Kingdom and Australia [48], which may have introduced variability in country contexts. While we excluded data explicitly linked to Australian contributors to mitigate this risk, some aggregated data may reflect mixed contexts.

### Conclusions

There is preliminary evidence that DMHIs can be delivered to people experiencing psychosis in a trial. Where assessed, interventions were generally delivered as intended and often reached participants considered representative of existing psychosis services. DMHIs may reduce symptoms by modifying users’ habitual behavioral and cognitive responses, yet mechanistic pathways require further validation. Implementation strategies aimed to improve the fit between DMHIs and users through flexible delivery and stakeholder involvement, and support DMHI use and delivery in trials through professional support and staff training. While these approaches appear promising, engagement with DMHIs varied between users, and the effectiveness of implementation strategies remains largely untested. Contextual factors often differed from stakeholder expectations, relating to users’ everyday factors outside of therapy, treatment expectations, or symptoms, rather than assumed technology-related issues, highlighting the need to address misconceptions and clearly communicate delivery requirements. Process evaluation remains underused; embedding it in future trials is a priority and could clarify mechanisms, guide implementation, and support the translation of effective DMHIs from trials to practice.

## Data Availability

The datasets generated or analyzed during this study are available from the corresponding author on reasonable request.

## Appendix

Multimedia Appendix 1 Completed review reporting checklists.

Multimedia Appendix 2 Review supplementary materials.

## Abbreviations

DMHI: digital mental health intervention
ERIC: Expert Recommendations for Implementing Change
MRC: Medical Research Council
NHS: National Health Service
PRISMA-ScR: Preferred Reporting Items for Systematic Reviews and Meta-Analyses extension for Scoping Reviews
PROSPERO: International Prospective Register of Systematic Reviews
RCT: randomized controlled trial
VR: virtual reality
