# Supplementary material for: Process Evaluation of Digital Mental Health Interventions for Psychosis: Scoping Review With Framework Synthesis
Source: JMIR Hum Factors. 2026 Jul 23;13:e88032. doi: 10.2196/88032 (PMC13448200; doi:10.2196/88032)
Supplement: Supplementary file 2 [file humanfactors_v13i1e88032_app2.docx]

**Multimedia Appendix 2: Supplementary Materials**

Contents

[Supplementary Methods SM1. Search terms and database list.	2](#_Toc213848363)

[Supplementary Table S2. Index randomised controlled trial risk of bias scores (N=14).	6](#_Toc213848364)

[Supplementary Table S3. Index randomised controlled trial summary characteristics (N=14).	7](#_Toc213848365)

[Supplementary Table S4. Process evaluation summary characteristics (N=45).	11](#_Toc213848366)

[Supplementary Results SR1. Coding tree	19](#_Toc213848367)

# Supplementary Methods SM1. Search terms and database list.

**Overview of database searches**

Articles in search phase 1 were identified via a database search of two databases (CENTRAL and PsycInfo) conducted on 31/01/2024 and were updated 22/10/2025. The search strategy included three concepts: psychosis, DMHIs, and RCTs (including abbreviations, synonyms and derivatives). Both medical subject headings and free-text words were used. Searches were restricted to peer-reviewed articles, but did not have date or language restrictions.

**Supplementary Table 1. Search strategy: participants, intervention, study**

| Type of term | Term | Keywords | MESH^a^ term |
| --- | --- | --- | --- |
|  |  |  |  |
| Participants | Psychosis | Psychos*, psychot*, schizo*, delusion*, hallucin* | Psychosis |
|  |  |  |  |
| Intervention | Digital intervention | Digital intervention, smartphone intervention, mobile intervention, internet intervention, web intervention, smartphone application, mobile application, telecare, etherapy, VR, virtual reality | Digital mental health intervention |
|  |  |  |  |
| Study | Randomised controlled trial | RCT, randomi* | Randomised controlled trial |
|  |  | NOT protocol |  |

^a^Medical subject heading.

**PsycInfo search strategy**

1. **Initial search (conducted 31/01/2024)**

**176** Results for ((((((**MeSH**: (randomized controlled trials))) *OR* ((**title**: ("RCT")) *OR* (**title**: ("randomi*"))) *OR* ((**abstract**: ("RCT")) *OR* (**abstract**: ("randomi*"))) *OR* ((**KEYWORDS**: ("RCT")) *OR* (**KEYWORDS**: ("randomi*")))) *NOT* (**title**: ("protocol")) *NOT* (**abstract**: ("protocol")) *NOT* (**Keywords**: ("protocol"))) *AND* (((**MeSH**: (psychosis)) *OR* (**title**: ("psychos*") *OR* **title**: ("psychot*") *OR* **title**: ("schizo*") *OR* **title**: ("delusion*") *OR* **title**: ("hallucin*")) *OR* (**abstract**: ("psychos*") *OR* **abstract**: ("psychot*") *OR* **abstract**: ("schizo*") *OR* **abstract**: ("delusion*") *OR* **abstract**: ("hallucin*")) *OR* (**Keywords**: ("psychos*") *OR* **Keywords**: ("psychot*") *OR* **Keywords**: ("schizo*") *OR* **Keywords**: ("delusion*") *OR* **Keywords**: ("hallucin*"))) *AND* (((**MeSH**: (digital mental health intervention)) *OR* (**title**: ("Digital intervention") *OR* **title**: ("smartphone intervention") *OR* **title**: ("mobile intervention") *OR* **title**: ("internet intervention") *OR* **title**: ("web intervention") *OR* **title**: ("smartphone application") *OR* **title**: ("mobile application") *OR* **title**: ("telecare") *OR* **title**: ("etherapy") *OR* **title**: ("VR") *OR* **title**: ("virtual reality")) *OR* (**abstract**: ("Digital intervention") *OR* **abstract**: ("smartphone intervention") *OR* **abstract**: ("mobile intervention") *OR* **abstract**: ("internet intervention") *OR* **abstract**: ("web intervention") *OR* **abstract**: ("smartphone application") *OR* **abstract**: ("mobile application") *OR* **abstract**: ("telecare") *OR* **abstract**: ("etherapy") *OR* **abstract**: ("VR") *OR* **abstract**: ("virtual reality")) *OR* (**Keywords**: ("Digital intervention") *OR* **Keywords**: ("smartphone intervention") *OR* **Keywords**: ("mobile intervention") *OR* **Keywords**: ("internet intervention") *OR* **Keywords**: ("web intervention") *OR* **Keywords**: ("smartphone application") *OR* **Keywords**: ("mobile application") *OR* **Keywords**: ("telecare") *OR* **Keywords**: ("etherapy") *OR* **Keywords**: ("VR") *OR* **Keywords**: ("virtual reality"))) *AND* **Peer-Reviewed Journals only**

2. **Updated search (conducted 22/10/2025)**

**0** Results for ((((((**MeSH**: (randomized controlled trials))) *OR* ((**title**: ("RCT")) *OR* (**title**: ("randomi*"))) *OR* ((**abstract**: ("RCT")) *OR* (**abstract**: ("randomi*"))) *OR* ((**KEYWORDS**: ("RCT")) *OR* (**KEYWORDS**: ("randomi*")))) *NOT* (**title**: ("protocol")) *NOT* (**abstract**: ("protocol")) *NOT* (**Keywords**: ("protocol"))) *AND* (((**MeSH**: (psychosis)) *OR* (**title**: ("psychos*") *OR* **title**: ("psychot*") *OR* **title**: ("schizo*") *OR* **title**: ("delusion*") *OR* **title**: ("hallucin*")) *OR* (**abstract**: ("psychos*") *OR* **abstract**: ("psychot*") *OR* **abstract**: ("schizo*") *OR* **abstract**: ("delusion*") *OR* **abstract**: ("hallucin*")) *OR* (**Keywords**: ("psychos*") *OR* **Keywords**: ("psychot*") *OR* **Keywords**: ("schizo*") *OR* **Keywords**: ("delusion*") *OR* **Keywords**: ("hallucin*"))) *AND* (((**MeSH**: (digital mental health intervention)) *OR* (**title**: ("Digital intervention") *OR* **title**: ("smartphone intervention") *OR* **title**: ("mobile intervention") *OR* **title**: ("internet intervention") *OR* **title**: ("web intervention") *OR* **title**: ("smartphone application") *OR* **title**: ("mobile application") *OR* **title**: ("telecare") *OR* **title**: ("etherapy") *OR* **title**: ("VR") *OR* **title**: ("virtual reality")) *OR* (**abstract**: ("Digital intervention") *OR* **abstract**: ("smartphone intervention") *OR* **abstract**: ("mobile intervention") *OR* **abstract**: ("internet intervention") *OR* **abstract**: ("web intervention") *OR* **abstract**: ("smartphone application") *OR* **abstract**: ("mobile application") *OR* **abstract**: ("telecare") *OR* **abstract**: ("etherapy") *OR* **abstract**: ("VR") *OR* **abstract**: ("virtual reality")) *OR* (**Keywords**: ("Digital intervention") *OR* **Keywords**: ("smartphone intervention") *OR* **Keywords**: ("mobile intervention") *OR* **Keywords**: ("internet intervention") *OR* **Keywords**: ("web intervention") *OR* **Keywords**: ("smartphone application") *OR* **Keywords**: ("mobile application") *OR* **Keywords**: ("telecare") *OR* **Keywords**: ("etherapy") *OR* **Keywords**: ("VR") *OR* **Keywords**: ("virtual reality"))) *AND* **Peer-Reviewed Journals only**

**Cochrane Library search strategy**

1. **Initial search (conducted 31/01/2024)**

ID	Search

#1	MeSH descriptor: [Randomized Controlled Trial] explode all trees

#2	RCT or randomis*

#3	protocol

#4	#1 OR #2

#5	#4 NOT #3

#6	MeSH descriptor: [Psychotic Disorders] explode all trees

#7	psychos* OR psychot* OR schizo* OR delusion* OR hallucin*

#8	#6 OR #7

#9	"digital mental health intervention" OR "Digital intervention" OR "smartphone intervention" OR "mobile intervention" OR "internet intervention" OR "web intervention" OR "smartphone application" OR "mobile application" OR telecare OR etherapy OR VR OR "virtual reality"

#10	#4 AND #8 AND #9

2. **Updated search (conducted 22/10/2025)**

ID	Search	Hits

#1	MeSH descriptor: [Randomized Controlled Trial] explode all trees	34

#2	RCT or randomis*	259950

#3	protocol	163793

#4	#1 OR #2	259982

#5	#4 NOT #3	217288

#6	MeSH descriptor: [Psychotic Disorders] explode all trees	4113

#7	psychos* OR psychot* OR schizo* OR delusion* OR hallucin*	83332

#8	#6 OR #7	83332

#9	"digital mental health intervention" OR "Digital intervention" OR "smartphone intervention" OR "mobile intervention" OR "internet intervention" OR "web intervention" OR "smartphone application" OR "mobile application" OR telecare OR etherapy OR VR OR "virtual reality"	20099

#10	#4 AND #8 AND #9 with Cochrane Library publication date Between Feb 2024 and Oct 2025	117

# Supplementary Table S2. Index randomised controlled trial risk of bias scores (N=14).

| **Study ID** | **Intervention name** | **Randomization process** | **Deviations from intended interventions** | **Missing outcome data** | **Measurement of the outcome** | **Selection of the reported result** | **Overall Bias** |
| --- | --- | --- | --- | --- | --- | --- | --- |
| Bucci (2018) | Actissist | + | ! | + | + | + | ! |
| Bucci (2024) | Actissist 2.0 | + | + | + | + | + | + |
| Cella (2022) | V-NeST | + | + | + | + | + | + |
| Craig (2018) | AVATAR1 | + | + | + | + | + | + |
| Dodgson (2025) | MUSE-ARMS | + | ! | ! | + | + | ! |
| Dudley (2024) | MUSE-FEP | + | + | + | + | + | + |
| Freeman (2016) | - | + | ! | + | ! | + | ! |
| Freeman (2022) | gameChange | + | ! | + | + | + | ! |
| Freeman (2023) | THRIVE | + | + | + | ! | + | ! |
| Garety (2021) | SloMo | + | + | + | + | + | + |
| Garety (2024) | AVATAR2 | + | + | + | + | + | + |
| Gumley (2022) | EMPOWER | + | + | + | ! | + | ! |
| Leff (2013) |  | ! | ! | + | ! | + | ! |
| Yiend (2023) | CBM-pa | + | + | + | + | + | + |
|  |  |  |  |  |  |  |  |
| Cell | Bias rating |  |  |  |  |  |  |
| + | Low |  |  |  |  |  |  |
| ! | Some |  |  |  |  |  |  |
| - | High |  |  |  |  |  |  |

# Supplementary Table S3. Index randomised controlled trial summary characteristics (N=14).

| First author (year) | Trial name | Trial phase using author phrasing | Recruitment locale(s) | Intervention group N (female) | Intervention group age M (SD) | Intervention group diagnoses (N) | Intervention group ethnicities (N) | Intervention description | Trial outcome | Risk of bias |
| --- | --- | --- | --- | --- | --- | --- | --- | --- | --- | --- |
|  |  |  | Recruitment source(s) |  |  |  |  |  |  |  |
| Bucci  (2018) | Actissist | Proof-of-concept | Northwest (England) | 24 (9) | Not stated | First episode psychosis (24) | White British/Irish (21)  Black Caribbean/ African (2)  Asian (1) | Smartphone-based intervention using a cognitive model of psychosis for key psychosis domains with both prompted and on demand use and to be delivered in any setting with technical support from the research team. | Positive | Some |
|  |  |  | NHS^a^ trusts |  |  |  |  |  |  |  |
| Bucci  (2024) | Actissist 2.0 | Efficacy | Unclear (England) | 87 (32) | 29.2 (9.2) | Schizophrenia spectrum disorders (87) | White/Caucasian (62)  Mixed/Multiple Ethnic Groups (5)  Asian/Asian British (13)  Black/African/Caribbean/Black British (6)  Arabic (1) | Smartphone-based intervention for early psychosis using cognitive behavioural therapy for psychosis principles to target maintaining cognitive appraisals. Users self-select content relating to key relapse risk indicators for psychosis. Includes both prompted and on demand use and can be delivered in any setting. | Negative | Low |
|  |  |  | CMHTs^b^ |  |  |  |  |  |  |  |
| Cella  (2022) | V-NeST^c^ | Pilot, feasibility | London (England) | 15 (not stated) | Not stated | Schizophrenia (not stated)  Documented episode of psychosis (not stated) | Not stated | Virtual reality cognitive behavioural therapy and cognitive remediation intervention for negative symptoms of schizophrenia delivered as scheduled sessions with support from graduate level psychologists. | Not applicable | Low |
|  |  |  | CMHTs |  |  |  |  |  |  |  |
| Craig (2018) | AVATAR^d^1 | Not stated | London (England) | 75 (18) | 42.5 (10.1) | Paranoid schizophrenia (57)  Schizoaffective disorder (8)  Unspecified psychosis (5)  Bipolar disorder (1)  Depression (4) | White British (26)  Black British (15)  Black Caribbean (7)  Black African (7)  Asian Indian (2)  Other (18) | Computer-based intervention using the principles of cognitive behavioural therapy for auditory verbal hallucinations delivered as scheduled sessions in clinical settings with support from experienced clinicians. | Positive | Low |
|  |  |  | NHS trusts  Self-referral |  |  |  |  |  |  |  |
| Dodgson (2025) | MUSE^e^ | Feasibility | Northeast (England) | 46 (29) | 19.0 (4.6) | Not applicable | White British (42)  White – Any other White Background (1)  Asian/Asian British (1)  Black/African/Caribbean/Black British – African (1)  Other (1) | Computer-based intervention targeting causal cognitive processes by providing psychoeducation to those at-risk of psychosis to address unusual sensory experiences, delivered remotely and as scheduled sessions with on-demand access by experienced clinicians. | Positive (functioning score at 12 weeks post-treatment)  Negative (auditory hallucinations at 12 weeks post-treatment) | Some |
|  |  |  | NHS trusts |  |  |  |  |  |  |  |
| Dudley (2024) | MUSE | Exploratory | Northeast (England) | 40 (18) | 31.65 (10.4) | First episode psychosis (40) | White (39)  Asian (1) | Computer-based intervention targeting causal mechanisms for hallucinations delivered as scheduled sessions by care coordinators in clinical settings or participants’ homes. | Not applicable | Low |
|  |  |  | NHS trusts |  |  |  |  |  |  |  |
| Freeman (2016) | None given | Not stated | Oxford  (England) | 15 (5) | 42.1 (13.4) | Schizophrenia (10)  Schizoaffective disorder (2)  Psychosis NOS^f^ (3) | White (14)  Mixed (1) | Virtual reality-based cognitive therapy for persecutory delusions delivered as scheduled sessions at the investigational study site with support setting up from a clinical psychologist. | Positive | Some |
|  |  |  | NHS Trusts CMHTs |  |  |  |  |  |  |  |
| Freeman (2022) | gameChange | Efficacy | Bristol Manchester Newcastle Nottingham Oxford  (England) | 174 (58) | 36.6 (12.8) | Schizophrenia (75)  Schizotypal disorder (1)  Delusional disorder (2)  Brief psychotic disorders (5)  Schizoaffective disorder (15)  Other psychotic disorder (2)  Unspecified psychosis (62) | White (152)  Black British (1)  Black African (1)  Black Other (1)  Pakistani (3)  Other (16) | Virtual reality-based cognitive therapy for agoraphobic avoidance and distress delivered as scheduled sessions in a clinical setting or participant homes with technical, logistical and emotional support from a mental health worker (peer support worker, assistant psychologist or clinical psychologist). | Positive | Some |
|  |  |  | NHS trusts CMHTs  Inpatient hospitals |  |  |  |  |  |  |  |
| Freeman (2023) | THRIVE^g^ | Not stated | Berkshire  Milton Keynes Northamptonshire  Oxford  (England) | 39 (14) | 41.1 (13.4) | Schizophrenia (20)  Schizoaffective disorder (5)  Delusional disorder (2)  Psychosis NOS (12) | White (28)  Black Caribbean (3)  Black African (2)  Black Other (1)  Indian (1)  Pakistani (2)  Chinese (1)  Other (1) | Virtual reality-based cognitive therapy for persistent persecutory delusions delivered as scheduled sessions in a clinical setting or participants’ homes with technical and emotional support from an assistant or clinical psychologists. | Negative | Some |
|  |  |  | NHS trusts |  |  |  |  |  |  |  |
| Garety (2021) | SloMo | Not stated | London  Oxford  Sussex  (England) | 181 (49) | 43.1 (11.7) | Schizophrenia (116) Schizoaffective disorder (30)  Delusional disorder (3)  Psychosis (other, 32) | White (120)  Black Caribbean (9)  Black African (12)  Black Other (16)  Indian (0)  Pakistani (4)  Chinese (1)  Other (19)  Missing (0) | *Intervention 1*. Web-based intervention for paranoia using cognitive behavioural therapy designed as a digital adjunct to sessions with trained doctoral-level psychologists delivered in a clinical setting or a participants’ homes.  *Intervention 2*. Smartphone-based intervention for paranoia using cognitive behavioural therapy designed for on-demand use in any setting without any support. | Negative  (Primary outcome)  Positive  (Secondary paranoia outcomes) | Low |
|  |  |  | CMHTs |  |  |  |  |  |  |  |
| Garety (2024) | AVATAR2 | Efficacy | South London  North London  Manchester  (England)  Glasgow (Scotland) | *AV-BRF*^h^*:* 116 (43)  *AV-EXT*^i^*:* 114 (42) | *AV-BRF:* 39.35 (13.31)  *AV-EXT:* 40.81 (13.69) | *AV-BRF:*  Schizophrenia-spectrum disorder (101)  Bipolar affective disorder (1)  Severe depressive episode with psychotic symptoms (14)  Not available/not applicable (0)  *AV-EXT:*  Schizophrenia-spectrum disorder (101)  Bipolar affective disorder (4)  Severe depressive episode with psychotic symptoms (8)  Not available/not applicable (1) | *AV-BRF:*  White (63)  Black or mixed Black (19)  South Asian or mixed South Asian (9)  Other (25)  *AV-EXT:*  White (71)  Black or mixed Black (19)  South Asian or mixed South Asian (6)  Other (18) | Computer-based intervention using the principles of cognitive behavioural therapy for auditory verbal hallucinations delivered as scheduled sessions in clinical settings or remotely by experienced clinicians. Two versions were tested: AV-BRF and AV-EXT (respectively 6 and 12 individual sessions). | Positive (distress at 16 weeks after baseline)  Negative (distress at 28 weeks after baseline) | Low |
|  |  |  | Mental health services |  |  |  |  |  |  |  |
| Gumley (2022) | EMPOWER^j^ | Feasibility | Glasgow (Scotland) | 42 (21) | 42.0 (13.0) | Schizophrenia (not stated)  Unspecified schizophrenia (not stated)  Delusional disorder (not stated)  Schizophreniform disorder (not stated)  Schizoaffective disorder (not stated) | Scottish (21)  Other British (1)  Other White (1)  Mixed (1)  Pakistani (2)  Indian (1)  African (3)  Unknown (0) | Smartphone-based intervention using the cognitive interpersonal model for psychosis for relapse prevention to be accessed in any setting in response to intervention prompts with technical and therapeutic support from clinical staff in a variety of roles (mental health nurse or clinical psychologist). | Not applicable | Some |
|  |  |  | CMHTs |  |  |  |  |  |  |  |
| Leff (2013) | None given | Proof-of-concept | London (England) | 26 (10) | Not stated | Not stated | Not stated | Computer-based intervention for auditory hallucinations delivered as scheduled sessions with technical and emotional support from a therapist. | Positive | Some |
|  |  |  | NHS trusts  CMHTs |  |  |  |  |  |  |  |
| Yiend (2023) | CBM-pa^k^ | Feasibility | London (England) | 32 (10) | 43.7 (9.8) | Schizophrenia (20)  Schizoaffective disorder (2)  Schizophreniform disorder (1)  Major depressive disorder (4)  Bipolar disorder (1)  Anxiety (2) | White (16)  Black (13)  Asian (2)  Mixed and other (1) | Computer-based cognitive bias modification intervention for paranoia delivered as scheduled sessions at the investigational study site with support from trained research workers. | Positive | Low |
|  |  |  | Investigational study site  NHS trusts  Self-referral Charity, peer support and service user networks |  |  |  |  |  |  |  |

^a^NHS: National Health Service.

^b^CMHT: Community Mental Health Team.

^c^V-NeST: Virtual Reality Supported Therapy for the Negative Symptoms of Schizophrenia.

^d^AVATAR: Audio Visual Assisted Therapy Aid for Refractory auditory hallucinations

^e^MUSE: Managing Unusual Sensory Experiences

^f^NOS: Not otherwise specified.

^g^THRIVE: Therapeutic Realistic Immersive Virtual Environments

^h^AV-BRF: AVATAR-Brief

^i^AV-EXT: AVATAR-Extended

^j^EMPOWER: Early signs Monitoring to Prevent relapse in psychosis and prOmote Wellbeing, Engagement, and Recovery.

^k^CPM-pa: Cognitive Bias Modification for paranoia

# Supplementary Table S4. Process evaluation summary characteristics (N=45).

| Study identification number | First author (year) | Associated paper/trial index paper | Process evaluation aim | Process evaluation scope | Methodology | Process evaluation participants/ contributors  (N, female) | Process evaluation participants/ contributors ethnicity (N) | Use of process evaluation label |
| --- | --- | --- | --- | --- | --- | --- | --- | --- |
|  |  |  |  |  | Data collection methods |  |  |  |
| 1.0 | Freeman (2022a) | Trial index paper | We aimed to evaluate the efficacy of an automated VR^a^ cognitive therapy (gameChange) to treat avoidance and distress in patients with psychosis, and to analyse how and in whom it might work. | Study intervention | Quantitative | Trial participants  (346, 111) | White (294)  Black British (2)  Black African (3)  Black Caribbean (4)  Black other (1)  Indian (2)  Pakistani (6)  Other (33)  Missing (1) | No |
|  |  |  |  |  | Trial data |  |  |  |
| 1.1 | Bond (2023) | Associated paper | We set out to use a peer research approach to explore participants’ experiences with gameChange VR therapy. This in-depth experiential exploration of user experience may inform the implementation in clinical services and future VR therapy development. | Study intervention  Intervention implementation | Qualitative | Intervention users  (20, 11) | White British (17)  British Asian (2)  Polish (1) | No |
|  |  |  |  |  | Interviews |  |  |  |
| 1.2 | Brown (2022) | Associated paper | The aim of this study is to explore the views of patients and staff on the provision of VR therapy on psychiatric wards. | Intervention implementation | Qualitative | Intended intervention users  (19, 7)  Ward staff  (22, 19) | Not stated | No |
|  |  |  |  |  | Interviews |  |  |  |
| 1.3 | Freeman (2022b) | Associated paper | In this paper we focus on the potential importance of severe agoraphobic avoidance and its treatment in patients with psychosis. | Study intervention | Quantitative | Trial participants  (345, 78) | White (293)  Black British (2)  Black African (3)  Black Caribbean (4)  Indian (2)  Black other (1)  Chinese (0)  Pakistani (6)  Other (33) | No |
|  |  |  |  |  | Trial data |  |  |  |
| 1.4 | Freeman (2023) | Associated paper | We assessed the experience with one such therapy of patients diagnosed with psychosis, including satisfaction, side effects, and positive experiences of access to the technology. We tested whether side effects affected therapy. | Study intervention | Quantitative | Intervention users  (122, 39) | White (105)  Black British (1)  Black African (1)  Black Caribbean (0)  Indian (0)  Black other (1)  Pakistani (2)  Other (12) | No |
|  |  |  |  |  | Trial data |  |  |  |
| 1.5 | Knight (2021) | Associated paper | The structures put in place to support the different disciplines working together on the design, particularly people with lived experience of psychosis, are detailed in this paper, with examples of how decisions were made and their outcomes. | Study intervention | Qualitative | LEAP^b^ group  (11, Not stated)  Workshop attendees (Not stated)  Research team  (Not stated)  Design group  (Not stated) | Not stated | No |
|  |  |  |  |  | Author description |  |  |  |
| 1.6 | Lambe  (2020) | Associated paper | This paper describes the process of development of an automated VR cognitive therapy targeting anxious avoidance of everyday social situations by patients with psychosis. | Study intervention | Mixed methods | People with lived experience  (53, not stated)  LEAP group  (10, not stated)  Clinical psychologists (Not stated)  Designers  (Not stated)  Software developers (Not stated) | Not stated | No |
|  |  |  |  |  | Author description  Stakeholder feedback  Usability testing |  |  |  |
| 2.0 | Gumley (2022) | Trial index paper | We aimed to establish the feasibility of undertaking a definitive randomised  controlled trial to determine the effectiveness of a blended digital intervention for relapse prevention in schizophrenia. | Study intervention | Quantitative | Trial participants  (73, 36) | Scottish (21)  Other British (1)  Other White (1)  Mixed (1)  Pakistani (2)  Indian (1)  African (3)  Unknown (0) | No |
|  |  |  |  |  | Trial data |  |  |  |
| 2.1 | Allan (2019) | Associated paper | This study aimed to discover and theoretically frame implementation expectations in advance of testing and synthesize these data into a framework. | Intervention implementation | Qualitative | Mental health staff  (54, not stated)  Intended users  (5, not stated)  Carers of intended users  (20, not stated) | Not stated | No |
|  |  |  |  |  | Focus groups |  |  |  |
| 2.2 | Allan (2021) | Associated paper | The primary aim of this study was to understand recruitment from the point of view of trial staff and discover what they consider important. | Trial procedures | Qualitative | Trial staff  (6, not stated) | Not stated | Yes |
|  |  |  |  |  | Trial data  Focus groups |  |  |  |
| 2.3 | Allan (2023) | Associated paper | We aimed to explore implementation behaviours that occurred in the EMPOWER^c^ feasibility trial from the point of view of carers, patients and mental health staff through interviews. | Intervention implementation | Qualitative | Intervention users (16)  Mental health staff (6)  Carers (1) | Not stated | Yes |
|  |  |  |  |  | Interviews |  |  |  |
| 3.0 | Garety (2021) | Trial index paper | To investigate the effects on paranoia and mechanisms of action of SlowMo, a digitally supported reasoning intervention, plus usual care compared with usual care only. | Study intervention | Quantitative | Trial participants  (361, 109) | White (120)  Black Caribbean (9)  Black African (12)  Black Other (16)  Indian (0)  Pakistani (4)  Chinese (1)  Other (19)  Missing (0) | No |
|  |  |  |  |  | Trial data |  |  |  |
| 3.1 | Greenwood (2022a) | Associated paper | The current evaluation aimed to investigate the impact of Patient and Public Involvement (PPI)^d^ in the SlowMo study. | Study intervention | Mixed methods | PPI members (9, 6) | White British (6)  BAME^e^ (3) | No |
|  |  |  |  |  | Author description  Trial data  Interviews |  |  |  |
| 3.2 | Greenwood (2022b) | Associated paper | This study explored the subjective service-user experience of the SlowMo therapy content and design; the experience of the blended therapy approach, including the triangle of the therapeutic alliance; and the experience of the digital aspects of the intervention. | Study intervention  Intervention implementation | Qualitative | Intervention users  (22, 4) | White British (18)  Other (not stated) | No |
|  |  |  |  |  | Interviews |  |  |  |
| 3.3 | Hardy (2018) | Associated paper | This study aimed to optimize the usability of an existing psychological intervention, Thinking Well, which targets reasoning processes in paranoia using a basic digital interface. | Study intervention | Mixed methods | Intended intervention users  (12, 7) | White British (111)  Black African (13)  Black other (13)  Black Caribbean (8)  Asian (6)  Mixed heritage not stated (6)  Arab (5)  Chinese (4)  Hispanic (2) | No |
|  |  |  |  |  | Author description  Trial data  Interviews |  |  |  |
| 3.4 | Hardy (2022) | Associated paper | This study explores the “digital divide” and mobile app engagement in the SlowMo randomized controlled trial. | Study intervention  Intervention implementation | Quantitative | Intervention users  (168, not stated) | White British (7)  Black British (2)  Black African (1)  Afro-Caribbean (1)  Black Caribbean and white (1) | No |
|  |  |  |  |  | Trial data |  |  |  |
| 3.5 | Hardy (2024) | Associated paper | This paper provides an overview of evidence-based co-design. We use the example of SloMo, a blended digital therapy for paranoia. We describe our transdisciplinary team collaboration and how this facilitates inclusive lived experience involvement. | Study intervention | Qualitative | Clinical academics (Not stated)  Designers/Industry team members (Not stated)  PPI colleagues (Not stated)  LEAP (Not stated) | Not stated | No |
|  |  |  |  |  | Author description |  |  |  |
| 3.6 | Ward (2022) | Associated paper | This paper provides a comprehensive  account of therapy personalisation within this targeted approach. | Study intervention | Mixed methods | Intervention users  (181, 49) | White (120)  Black Caribbean (9)  Black African (12)  Black Other (16)  Indian (0)  Pakistani (4)  Chinese (1)  Other (19) | No |
|  |  |  |  |  | Trial data |  |  |  |
| 4.0 | Bucci (2018) | Trial index paper | This study assesses the feasibility and acceptability of Actissist, a digital health intervention grounded in the cognitive model of psychosis that targets key early psychosis domains. | Study intervention | Mixed methods | Trial participants  (36, 18) | White British/Irish (31)  Black Caribbean/ African (4)  Asian (1) | No |
|  |  |  |  |  | Trial data  Participant feedback |  |  |  |
| 4.1 | Berry (2020) | Associated paper | This article describes how our multidisciplinary team designed and developed Actissist. This article describes: (i) how Actissist was informed by initial qualitative interviews and focus groups and an expert reference group; (ii) refinements made to the app based on ongoing user feedback; (iii) successes and challenges encountered; and (iv) learning points and recommendations for involving stakeholders in digital health interventions. | Study intervention | Qualitative | Academics  (2, not stated)  Clinicians  (65, not stated)  Individuals with lived experience  (54, not stated)  Software engineers  (3, not stated) | White British (53)  Mixed (4)  White Irish (1)  Black British (1)  Missing (3) | No |
|  |  |  |  |  | Author description  Beta-tester feedback  Focus groups  Interviews |  |  |  |
| 4.2 | Bucci (2019) | Associated paper | The aim of the current study is to explore frontline staff views regarding the utility and appropriateness of using digital tools in the healthcare pathway for people accessing specialist secondary care mental health services. | Implementation | Qualitative | Mental health staff  (48, 27) | White British (40)  Mixed (4)  White Irish (1)  Missing (3) | No |
|  |  |  |  |  | Focus groups |  |  |  |
| 4.3 | Eisner (2023) | Associated paper | To examine how people with psychosis engaged with the cognitive behaviour therapy (CBT^f^)-informed Actissist app over a 12-week intervention period, and to examine factors associated with app engagement. | Study intervention | Quantitative | Intervention users  (24, 9) | White British/Irish (21)  Black Caribbean/ African (2)  Asian (1) | No |
|  |  |  |  |  | Trial data |  |  |  |
| 5.0 | Craig (2018) | Trial index paper | We aimed to investigate the effect of AVATAR^g^ therapy on auditory verbal hallucinations, compared with a supportive counselling control condition. | Study intervention | Quantitative | Trial participants  (150, 48) | White British (26)  Black British (15)  Black Caribbean (7)  Black African (7)  Asian Indian (2)  Other (18) | No |
|  |  |  |  |  | Trial data |  |  |  |
| 5.1 | Deamer (2023) | Associated paper | We build on previous research and question what it is about this innovative, interactional technique that might be contributing to the efficacy of this therapy? | Study intervention | Qualitative | Therapy completers  (8, not stated) | Not stated | No |
|  |  |  |  |  | Trial data |  |  |  |
| 5.2 | Hall (2018) | Associated paper | This study assessed the subjective experience of participating in a clinical trial, specifically positive and negative experiences and the experience of audio recording assessment sessions. | Trial procedures | Quantitative | Trial participants  (40, 13) | White British (12)  Black British (8)  Black Caribbean (4)  Black African (3)  Asian Indian (2)  Asian Chinese (1)  Other (8) | No |
|  |  |  |  |  | Trial data |  |  |  |
| 5.3 | O-Brien (2021) | Associated paper | We aim to (1) map relating behaviours between participants and their created avatars and (2) examine therapeutic actions delivered within AVATAR dialogue. | Study intervention | Mixed methods | Intervention completers  (25, not stated) | White British (9) Black (9)  Other (7) | No |
|  |  |  |  |  | Trial data |  |  |  |
| 5.4 | Rus-Calafell (2020) | Associated paper | The present study explores, for the first time, the contribution of sense of voice presence, together with session-by-session reduction of anxiety and paranoid attributions about the avatar, to changes in primary outcomes following AVATAR therapy. | Study intervention | Quantitative | Intervention users  (39, 9) | White British (13)  Black British (5)  Black Caribbean (3)  Black African (5)  Asian Indian (3)  Other (10) | No |
|  |  |  |  |  | Trial data |  |  |  |
| 5.5 | Rus-Calafell (2022) | Associated paper | We aimed to explore enablers and barriers to engagement with the therapy and potential for real-world impact on distressing voices. | Study intervention | Qualitative | Intervention users  (14, 4) | White British (6)  Black British (1)  Black Caribbean (2)  Black African (3)  Other (2) | No |
|  |  |  |  |  | Interviews |  |  |  |
| 5.6 | Ward 2020 | Associated paper | This article presents, for the first time, the full range of therapeutic targets along with information on acceptability and potential side effects. | Study intervention  Intervention implementation | Mixed methods | Intervention completers  (53, 12) | White British (17)  Black British/ Caribbean (14)  Other (22) | No |
|  |  |  |  |  | Trial data |  |  |  |
| 5.7 | Ward 2021 | Associated paper | This study aims to explore whether the extent of everyday voice engagement and participant–avatar interaction within AVATAR therapy are associated with the degree to which the person’s dominant voice is experienced as a highly characterised social agent. | Study intervention | Quantitative | Intervention users  (60, 15) | White British (23) Black British (8)  Black Caribbean (5)  Black African (7)  South Asian (2) Other (15) | No |
|  |  |  |  |  | Trial data |  |  |  |
| 6.0 | Yiend (2023) | Trial index paper | Study objectives were (i) test the intervention’s feasibility, (ii) provide effect size estimates, (iii) assess dose–response and (iv) select primary outcomes for future trials. | Study intervention  Trial procedures | Quantitative | Trial participants  (63, 21) | White (30)  Mixed and other (6)  Asian (5)  Black (21) | No |
|  |  |  |  |  | Trial data |  |  |  |
| 6.1 | Leung (2019) | Associated paper | We conducted a qualitative investigation of a novel intervention called CBM^h^ for paranoia (CBM-pa) to examine its acceptability in patients with psychosis. | Study intervention  Trial procedures | Qualitative | Intervention users  (8, 3) | Black British (2)  White British (2)  White Irish (1)  Pakistani (1)  Indian (1)  White and Asian (1) | No |
|  |  |  |  |  | Interviews |  |  |  |
| 7.0 | Cella (2022) | Trial index paper | This study tests the feasibility and acceptability of a novel Virtual Reality Supported Therapy for the Negative Symptoms of Schizophrenia (V-NeST). | Study intervention  Trial procedures | Mixed methods | Trial participants  (30, 9) | Not stated | No |
|  |  |  |  |  | Interviews  Trial data |  |  |  |
| 7.1 | Cella (2023) | Associated paper | This study evaluates, for the first time, the feasibility and acceptability of a novel Virtual Reality Supported Therapy for the Negative SympToms of Psychosis. | Study intervention  Trial procedures | Mixed methods | Trial participants  (30, 9)  Interview participants  (9, 3) | Not stated | No |
|  |  |  |  |  | Interviews  Trial data |  |  |  |
| 8.0 | Freeman 2023 | Trial index paper | We aimed to compare VR cognitive therapy with an alternative VR therapy (mental relaxation), with an emphasis on understanding potential mechanisms of action. | Study intervention | Quantitative | Trial participants  (80, 31) | White (64)  Black Caribbean (3)  Black African (2)  Black other (1)  Indian (1)  Pakistani (3)  Chinese (1)  Other (5) | No |
|  |  |  |  |  | Trial data |  |  |  |
| 9.0 | Leff (2013) | Trial index paper | To develop a computerised system that enables the patient to create an avatar of their persecutor. To encourage them to engage in a dialogue with the avatar, which the therapist is able to control so that the avatar progressively yields control to the patient. | Study intervention | Quantitative | Trial participants  (26, 10) | Not stated | No |
|  |  |  |  |  | Trial data |  |  |  |
| 9.1 | Leff (2014) | Associated paper | We discuss the possible psychological mechanisms for the success of Avatar therapy and the implications for the origins of persecutory voices. | Study intervention | Qualitative | Intervention users  (16, not stated) | Not stated | No |
|  |  |  |  |  | Author interpretation |  |  |  |
| 10.0 | Bucci (2024) | Trial index paper | This parallel, assessor-blinded, randomized clinical trial aimed to investigate whether a cognitive behaviour therapy-informed digital health intervention (Actissist app) confers added benefit on psychotic symptoms over and above remote symptom monitoring (ClinTouch app). | Study intervention  Trial procedures | Quantitative | Trial participants (172, 64) | White/Caucasian (88)  Mixed/multiple ethnic groups (8)  Asian/Asian British (27)  Black/African/Caribbean/Black British (11)  Arabic (2)  Other (3) | No |
|  |  |  |  |  | Trial data |  |  |  |
| 10.1 | Bucci (2025) | Associated paper | This qualitative study aimed to explore participants’ experiences with the Actissist app by analysing exit interviews from those who received the intervention during the Actissist 2.0 trial | Study intervention  Intervention implementation | Qualitative | Interviewed intervention users (21, 8) | White (17)  Mixed (1)  Asian (2)  Black (1) | No |
|  |  |  |  |  | Interviews |  |  |  |
| 10.2 | Hassan (2023) | Associated paper | This study describes and compares engagement patterns among participants in the two arms of the Actissist 2.0 randomised controlled trial. | Study intervention | Quantitative | Trial participants (172, 62) | White/Caucasian (88)  Mixed/multiple ethnic groups (8)  Asian/Asian British (27)  Black/African/Caribbean/Black British (11)  Arabic (2)  Other (3) | No |
|  |  |  |  |  | Trial data |  |  |  |
| 11.0 | Garety (2024) | Trial index paper | This randomized phase 2/3 trial assesses the efficacy of two forms of AVATAR therapy, AVATAR-Brief (AV-BRF) and  AVATAR-Extended (AV-EXT), both combined with treatment as usual (TAU) compared to TAU alone, and conducted an intention-to-treat analysis. | Study intervention | Quantitative | Trial participants (345, 129) | White (203)  Black or mixed Black (57)  South Asian or mixed South Asian (27)  Other (58) | No |
|  |  |  |  |  | Trial data |  |  |  |
| 11.1 | Orwid (2024) | Associated paper | This article describes a Patient and Public Involvement (PPI) led creative workshop space held within a clinical trial of a talking therapy for distressing voices (AVATAR2). | Study intervention | Qualitative | PPI colleagues (11, not stated)  Trial staff (7, not stated) | Not stated | No |
|  |  |  |  |  | Document analysis  Author description |  |  |  |
| 12.0 | Dodgson (2025) | Trial index paper | This study aimed to test the feasibility of  delivering MUSE^i^ within a RCT^j^ design. | Study intervention  Trial procedures | Quantitative | Trial participants (93, 54) | White British (85)  White – any other White background (2)  White Irish (1)  Asian/Asian British (3)  Black/African/Caribbean/Black British (1)  Other (1) | No |
|  |  |  |  |  | Trial data |  |  |  |
| 13.0 | Dudley (2024) | Trial index paper | The Managing Unusual Sensory Experiences for First-Episode-Psychosis (MUSE-FEP) trial aimed to establish the feasibility and acceptability of a brief, hallucination-specific, digitally provided treatment, delivered by a non-specialist workforce for people with psychosis. | Study intervention  Trial procedures | Quantitative | Trial participants (82, 38) | White (78)  Asian (2)  Black (1)  Mixed (1) | No |
|  |  |  |  |  | Trial data |  |  |  |
| 13.1 | Dudley (2025) | Associated paper | We evaluated if we can increase access to psychological therapy by using a brief treatment, focused only on understanding and dealing with hallucinations (Managing Unusual Sensory Experiences; MUSE), delivered by a less trained but more widely available workforce that harnessed the benefits (engaging content, standardisation) afforded by digital technology. | Study intervention  Intervention implementation | Mixed-methods | Intervention users (38,19) | White British (36)  White other (1)  Black British (1) | No |
|  |  |  |  |  | Trial data  Interviews |  |  |  |

^a^VR: Virtual reality.

^b^LEAP: Lived experience advisory panel.

^c^EMPOWER: Early signs Monitoring to Prevent relapse in psychosis and prOmote Wellbeing, Engagement, and Recovery.

^d^PPI: Patient and public involvement.

^e^BAME: Black, Asian and Minority ethnic.

^f^CBT: Cognitive behavioural therapy.

^g^AVATAR: Audio Visual Assisted Therapy Aid for Refractory auditory hallucinations

^h^CBM: Cognitive Bias Modification.

^i^MUSE: Managing Unusual Sensory Experiences

^j^RCT: Randomized Controlled Trial

# Supplementary Results SR1. Coding tree
